# Extracts of *Sida cordifolia* contain polysaccharides possessing immunomodulatory activity and rosmarinic acid compounds with antibacterial activity

**DOI:** 10.1186/s12906-022-03502-7

**Published:** 2022-01-27

**Authors:** Haroon Iqbal, Claire L. Wright, Sue Jones, Goncalo Rosas da Silva, John McKillen, Brendan F. Gilmore, Owen Kavanagh, Brian D. Green

**Affiliations:** 1 Institute for Global Food Security, School of Biological Sciences, Queen’s University Belfast, Belfast, BT9 5AG UK; 2 School of Pharmacy, Queen’s University Belfast, Belfast, BT9 7BL UK; 3 School of Science, Health & Technology, York St John University, York, YO31 7EX UK; 4 Veterinary Science Division, Agri-Food and Biosciences Institute, Stormont, Belfast, BT4 3SD UK

**Keywords:** Antibiotic resistance, Antibiotic alternative, Feed supplement, *Sida cordifolia*, Immunomodulation, Immunostimulant, Plant-derived biological response modifiers, Antimicrobial, *Galleria mellonella*, Polysaccharides, Rosmarinic acid

## Abstract

**Background:**

The overuse of antibiotics has led to increased antimicrobial resistance, but plant-derived biological response modifiers represent a potential alternative to these drugs. This investigation examined the immunomodulatory and antibacterial activities of *Sida cordifolia* (used in ethnomedicinal systems to treat infectious disease).

**Methods:**

Successive extractions were performed from the roots of these plants in hexane, chloroform, methanol and water. Immunomodulatory activity was determined in a series of experiments measuring the responses of splenocytes, macrophages and an in vivo model of innate immunity (*Galleria mellonella*)*.* Antibacterial activity was assessed by determining minimum inhibitory/bactericidal concentrations (MIC/MBCs) for various Gram-positive and Gram-negative bacterial strains.

**Results:**

Immunomodulatory activity was confined to the aqueous extract, and further fractionation and biochemical analysis yielded a highly potent polysaccharide-enriched fraction (SCAF5). SCAF5 is a complex mixture of different polysaccharides with multiple immunomodulatory effects including immune cell proliferation, antibody secretion, phagocytosis, nitric oxide production, and increased expression of pro-inflammatory cytokines. Furthermore, *Galleria mellonella* pre-treated with SCAF5 produced more haemocytes and were more resistant (*P* < 0.001) to infection with methicillin-resistant *Staphylococcus aureus* (MRSA) with a 98% reduction in bacterial load in pre-treated larvae compared to the negative control. The antibacterial activity of *Sida cordifolia* was confined to the methanolic fraction. Extensive fractionation identified two compounds, rosmarinic acid and its 4-O-β-d-glucoside derivative, which had potent activity against Gram-positive antibiotic-resistant bacteria, including MRSA.

**Conclusions:**

*Sida cordifolia* counters bacterial infections through a dual mechanism, and immunomodulatory polysaccharides from this plant should be isolated and characterised to realise their potential as anti-infective agents. Such properties could be developed as an antibiotic alternative (1) in the clinic and (2) alternative growth promoter for the agri-food industry.

**Supplementary Information:**

The online version contains supplementary material available at 10.1186/s12906-022-03502-7.

## Background

Antibiotics derived from fungi or bacteria (or synthetic derivatives) are one of the most important discoveries of the last century and are widely used for effectively treating human and animal disease [1]. These compounds also serve as prophylactic growth promoters in the domesticated livestock industry by reducing subclinical disease burden and/or reducing the number of metabolites produced by microbes which may act to depress growth [2]. However, due to their widespread overuse in medicine and the agri-food industry, antibiotic resistance among bacterial pathogens has emerged as one of the greatest threats to global public health [3, 4]. According to the World Health Organization (WHO), the global systematic misuse and overuse of these drugs in human medicine and food production has created an alarming level of antimicrobial resistance which could render antibiotic therapy redundant [5].

As antibiotic resistance exposes humans to potentially fatal infectious diseases, one approach has been a shift in focus to discovery of alternatives to these drugs. It is estimated that 25–50% of all medical drugs on the market were originally derived from plants, which have been shown to produce a plethora of antimicrobial secondary metabolites to protect themselves [6]. Furthermore, phytochemicals exhibiting antibacterial activity against antibiotic-resistant pathogenic bacteria have been extensively described elsewhere [6, 7]. This includes the polyphenol rosmarinic acid, isolated and identified in the present study, which has previously been shown to inhibit the growth of *Staphylococcus aureus* and prevent it from forming biofilms [8]. One of the most promising plant-derived alternatives to prophylactic antimicrobials since the emergence of widespread antibiotic resistance is the use of phytochemical immunomodulators such as exogenously-derived Biological Response Modifiers (BRMs) [9]. Immunomodulators are compounds that interact with the host immune system and up- or down-regulate specific immune responses. BRMs are chemically diverse and include a range of phytochemicals such as lectins, saponins and polysaccharides [10]. BRMs are postulated to instigate immunomodulatory responses by triggering one or more of the diverse range of pattern recognition receptors (PRRs) expressed by immune cells such as macrophages [9, 10]. Mammals and plants possess PRRs which sense infection by recognising signals called pathogen-associated molecular patterns (PAMPs) unique to each invading microorganism. Upon detection of a PAMP (e.g. Gram-negative bacteria lipopolysaccharide (LPS)), the PRR (in this example, a macrophage-associated Toll-like receptor 4 (TLR4)) triggers an array of antimicrobial innate and adaptive immune responses that eliminate the potential threat. Relatively few BRMs have been discovered thus far and there is merit in screening plant materials used to treat infectious disease. Polysaccharides extracted from certain shrubs and medicinal plants are known to trigger various immunological responses [11]. For example, a polysaccharide isolated from *Lycium barbarum* stimulates splenocyte proliferation and macrophages activation [12, 13]. In murine macrophage RAW 264.7 cells polysaccharides extracted from *Ganoderma sinese* promoted phagocytosis, release of NO and release cytokines such as IL-1α, IL-6, IL-10 and TNF-α [14].

For the current study *Sida cordifolia* L., a perennial subshrub in the Malvea tribe of the subfamily malvoideae (Malvaceae), was investigated. In India, the *Sida* genus is represented by circa 19 species of *Sida* of which the most widespread is *Sida cordifolia* [15]. The medicinal use of *Sida* in India can be traced back over 2000 years to the Charaka Samhita, where the roots were mixed with milk or honey (‘*vyādhikshamatva*’) to enhance immunity and in the treatment of ailments suggestive of infective disease [16, 17]. Similarly, in parts of Central America where it is known as Chichibe, *S. rhombifolia* was found to be used by the Mopan Maya of Belize and Guatemala to enhance immunity, and as an anti-infective agent for the treatment of wounds [18]. In Africa, species of *Sida* were used to treat infective diseases and ailments normally associated with the immune system including malarial fever, and it is currently used as an antimicrobial and anti-inflammatory agent [19, 20]. The widespread, international use of this species in different ethnomedical systems, as an anti-inflammatory, anti-pyretic, anti-infective and wound healing agent, and as an antidote to snake venom suggests this plant possesses immunomodulating and antimicrobial compounds.

Here, we investigated the in vitro and in vivo immunomodulating and antibacterial properties of *Sida cordifolia* and characterised some of its bioactive components. *Galleria mellonella* larvae were used as an invertebrate in vivo model as (i) it’s a commonly used in vivo invertebrate infection model to study the efficacy of antimicrobial drugs [21], and (ii) its innate immune system is similar to vertebrates, and, (iii) infection models have been shown to produce comparable data to animal studies [22]. An enriched aqueous fraction (referred to as SCAF5) possesses immunomodulatory activity. The fraction appears to be a complex mixture of polysaccharides, and their observed in vitro immunomodulating activities were verified in vivo in an established insect model of innate immunity. The plant also contained potent antibacterial activity which resided in the methanol fraction and had activity against antibiotic-resistant Gram-positive bacteria. Two antibacterial compounds were isolated and identified as rosmarinic acid and its derivative rosmarinic acid 4-O-β-d-glucoside.

## Methods

### Extraction

*Sida cordifolia* L radix was collected in Karnataka, India (2012), and was donated by Pukka Herbs, Bristol. A voucher specimen was deposited in the DBN Economic Collections, Glasnevin Herbarium Dublin (DBN 06:201261). Plant roots were washed with isopropanol and water and lyophilised roots were homogenised to a fine powder using an IKA® A11 analytical mill (IKA® Werke GmbH & Co. KG, Staufen, Germany). Powdered roots were macerated successively in n-hexane, chloroform, methanol and double-distilled water (ddH_2_O) at room temperature (24 h). N-hexane, chloroform and methanol extracts were concentrated by rotary evaporation (45 °C). Aqueous extract was centrifuged at 4000 *g* for 15 min and then lyophilised and the subsequent extract underwent ethanol (abs.) precipitation (1:4 v/v) overnight. The resulting precipitate was centrifuged (1000 *g;* 10 min) and the supernatant discarded, and the pellet was lyophilised.

### Aqueous crude precipitate fractionation

The crude aqueous precipitate obtained (2.0 g; 50 mg/ml) was further fractionated using a DEAE™ Sephadex A-50 column (GE Healthcare, Kent, UK) pre-equilibrated with ddH_2_0, then eluted stepwise into low, medium, and high ionic strength solutions (0, 0.75, 2 mol/l NaCl). After lyophilising, each of these were fractionated using 100 kDa molecular weight cut-off (MWCO) filters (Vivaspin, Sartorius, Gottingen, Germany) to obtain > 100 kDa and < 100 kDa fractions (SCAF0–5; supplementary data Table S1) where SCAF0 is the equivalent of the pre-fractionated aqueous crude precipitate. The protein content of SCAF fractions was determined (Bradford assay, Biorad, Hertfordshire, UK) and found to be negligible.

### Endotoxin contamination

The *Limulus* Amebocyte Lysate (LAL) gel clot assay (Pyrosate; Associates of Cape Cod, Inc., Liverpool, UK) was performed to detect Gram-negative bacterial endotoxin contamination to a high degree of sensitivity (0.03 endotoxin units (EU)/ml). A glucan blocker was used to eliminate false positives due to activation of clotting enzyme by plant cellulose. A solution of lyophilised aqueous extract (100 ng/ml) in sterile certified endotoxin-free water (Sigma) was incubated with LAL reagent and the assay performed in accordance with the manufacturer’s instructions.

### Immunological effects of SCAF fractions

#### Lymphocyte cell preparation

Spleens were aseptically removed from adult female Balb/C mice sacrificed by cervical dislocation and collected in 5 ml RPMI-1640 medium (ThermoFisher Scientific™, Paisley, UK) containing 1% penicillin-streptomycin. Cells were collected into fresh RPMI medium using sterile 40 μM nylon cell strainers (BD Falcon, Oxford). Cell suspensions were centrifuged (300 *g;* 10 min), the supernatant discarded, and erythrocytes depleted using a red blood cell lysis buffer (Red Blood Cell Lysing Buffer Hybri-Max, Sigma-Aldrich). Splenocytes were re-suspended in RPMI 1640-Glutamax medium containing 1% penicillin-streptomycin and 10% heat-inactivated foetal bovine sera (FBS). After cell counting (Countess® Automatic Cell Counter, Invitrogen) cells were seeded (2 × 10^5^ cells/well) into 96-well plates (BD Falcon).

#### Lymphocyte proliferation

Splenocyte proliferation was measured by adding AlamarBlue (10% v/v; Invitrogen, ThermoFisher Scientific, Paisley, UK) to each well for 24 h according to the manufacturer’s instructions, with optical density (OD; 570 nm) determined using a microplate reader (Safire2, Tecan, Switzerland). Concanavalin A (Con A) is a lectin which binds to mannose-containing receptors and is well characterised activator of murine lymphocytes [23].. SCAF0-SCAF5 diluted in RPMI medium (100 μl; 10 ng/ml–1 mg/ml) were tested in triplicate with (Con A; 5 μg/ml) (Sigma-Aldrich) as a positive control and supplemented media as a negative control. Results were expressed as the splenocyte proliferation index (OD treated cells divided by OD negative control cells).

#### Antibody secretion

Immunoglobulin (IgG) production was evaluated using an in-house sandwich enzyme-linked immunosorbent assay (ELISA). Splenocytes prepared as described above were incubated with extracts (48 h), 96-well plates were centrifuged (300 *g*; 10 min), supernatants collected by aspiration, and stored at − 20 °C until analysis. ELISA plates were coated overnight (4 °C) with goat anti-mouse IgG capture antibody (Sigma Aldrich) diluted 1:1000 (10 μg/ml; 75 μl/well) in 0.1 M sodium bicarbonate buffer (pH 9.4). Wells were blotted then blocked with 1% non-fat dried milk (Marvel; 2 h; 37 °C). After washing, diluted cell supernatants were added (75 μl; 2 h; 37 °C) to wells and incubated. Wells were washed with ELISA wash solution and 75 μl of goat anti-mouse IgG secondary detector antibody (HRP-conjugated; 1:2000; Sigma-Aldrich) was added (1 h; 37 °C). Plates were again washed and tetramethylbenzidine (TMB) solution (Sigma-Aldrich) was added, after 10 min H_2_SO_4_ (2.5 M) was added and absorbance (450 nm) measured.

#### Macrophage activation and phagocytosis

The murine macrophage cell line RAW264.7 (European Collection of Cell Cultures ECACC 91062702) was cultured in Dulbecco’s modified Eagle’s medium (DMEM), containing 10% heat inactivated FBS and 1% penicillin-streptomycin. Cells were grown to confluence in 75cm^2^ culture flasks in the presence of 5% CO_2_ at 37 °C. RAW 264.7 cells were cultured, seeded (1 × 10^4^ cells/well), and exposed to SCAF fractions (24 h; 37 °C). Macrophage phagocytosis was assessed by the uptake of neutral red (NR) dye [24]. Sterile homogenous NR solution (0.1% v/v) was added to cells in each well (6 h). Contents were then discarded, adherent cells were washed twice with PBS, lysed (deionised water with 50% absolute ethanol and 1% glacial acetic acid), incubated at room temperature (2 h), and optical densities measured (540 nm). Lipopolysaccharide (LPS; 1.0 μg/ml; Sigma-Aldrich) was used as a positive control and media as a negative control. Macrophage activation was determined by nitric oxide (NO) production as measured by the Griess assay [25] using a sodium nitrite standard curve which was performed in accordance with the manufacturer’s instructions (ThermoFisher Scientific, Paisley).

#### Measurement of cytokine expression

RAW 264.7 cells (as described above) were seeded into 6-well cell culture plates (5 × 10^5^ cells/well Corning® Costar, Sigma Aldrich). SCAF extracts were added to seeded wells (*n* = 3), cells were incubated (37 °C; 24 h), then removed using a cell scraper. Triplicate samples were pooled, the total number of cells was determined, and diluted to a density of 3 × 10^5^ cells/ml. Splenocytes were separately prepared at 1.5 × 0^6^ cells/ml. Cells were centrifuged (300 *g;* 15 min), and RNA isolated from cell pellets (RNeasy Mini kit; Qiagen LTD, Manchester, UK). Purity of isolated RNA was assessed to ensure 260/280 ratio ≥ 2.0 (Nanodrop 2000c; Thermo Scientific, Loughborough, UK). Complementary DNA (cDNA) was synthesised from RNA samples (stored at − 80 °C) using the Transcriptor First-Strand cDNA synthesis kit (Roche Diagnostics Ltd., Sussex, UK) (*n* = 3) according to the manufacturer’s instructions. RT-qPCR was performed on an Eppendorf Mastercycler (Eppendorf, Stevenage, UK) in accordance with the Minimum Information for Publication of Quantitative Real-Time PCR Experiments (MIQE) guidelines [26]. Hydroxymethylbilane synthase (HPRT1) was used as the reference gene for normalisation. Primer sequences for the selected cytokines measured are shown in Table S2 along with post-amplification melting curves showing reaction specificity (Fig. S1). RT-PCR products were resolved in 1.0% agarose gel electrophoresis. PCR reactions contained 100 ng cDNA and underwent 45 cycles of denaturation (94 °C; 15 s), annealing (58 °C; 60s) and extension (72 °C; 60s) using a LightCycler 480 system (Roche Diagnostics Ltd., Sussex, UK). The comparative cycle threshold (Ct) method was used to normalise the gene of interest to HPRT1 with treated samples (TRT) compared with untreated cells (CTL).

### Monosaccharide composition

SCAF0 and SCAF5 (100 mg) were hydrolysed in trifluoracetic acid (105 °C; 7 h 10 ml; 1 M; TFA; Sigma-Aldrich) and lyophilised. Samples (2 mg) were derivatised by silylation reactions for 12 h at room temperature with N,O-Bis (trimethylsilyl) trifluoroacetamide (500 μl; BSTFA) with 1% trimethylchrosilane in 1 ml anhydrous pyridine. GC-MS analysis of silylated hydolysates was performed using gas chromatography (Agilent 7890A) interfaced with a mass selective detector (Agilent 5975C), with a ZB semi-volatiles column (30 m × 0.25 mm × 0.25 μM Zebron, Phenomenex Inc.) with helium as the carrier gas at a constant rate of 1 ml/min. The injector and MS source temperatures were set at 260 °C and 230 °C, respectively. The column temperature program consisted of injection at 80 °C held for 1 min, with temperature increase of 15 °C/min to 300 °C, then held at 300 °C for 15 min. The MS was operated in the electron impact mode with ionisation energy of 70 eV. The scan range was set from mass scan range was 50–550 Da. Injection volume was 1 μl and the inlet had a split flow of 20 ml^− 1^ (split ratio 20:1). Data was acquired and processed with the ChemStation software (Hewlett Packard) and monosaccharide identification was performed by comparison of retention time and mass spectra against known standards and/or the NIST mass spectral library (National Institute of Standards and Technology, USA).

### Antimicrobial activity

Several Gram-positive and Gram-negative bacterial strains were selected for Minimum Inhibitory Concentration (MIC) and Minimum Bactericidal Concentration (MBC) assays. These were *P. aeruginosa* (PAO1: ATCC 47085), *S. epidermis* (NCTC 11964), *S. aureus* (NCTC 12493) (ATCC 43300), *E. coli* (ACTCC 11303), *A. baumannii* (NCTC 13304), *Enterococcus Faecalis* (DZMZ 25390), Methicillin-Resistant *Staphylococcus aureus* (MRSA: ATCC 43300) and Methicillin-Resistant *Staphylococcus epidermidis* (MRSE: NCTC 11964). The strains used in this study are type strains from several culture collections used for routine screening assays whose antimicrobial susceptibility profiles are well characterised [27–29].

For the determination of MIC, the strains were tested against increasing concentrations of n-hexane (SCHEX), chloroform (SCCL), methanol (SCMEX) and aqueous (SCAQ) crude extracts using broth microdilution following the performance standards as recommended by the Clinical and Laboratory Standards Institute (CLSI) [30]. Briefly, test strains were grown overnight at 37 °C in Mueller Hinton Broth (MHB) with agitation and the organism suspension adjusted to the density of 0.5 McFarland standard. The test compounds were tested at concentration range from 0.003 – 8000 μg/ml and the MIC determined as the lowest concentration (mmol) corresponding to absence of growth. Bactericidal activity was analysed by elucidation of the MBC where aliquots from each well showing no visible growth were plated onto a Mueller-Hinton agar plate. The agar plates were then incubated overnight at 37 °C and checked for a 99.9% kill to determine MBC.

### Isolation and characterisation of SC1 and SC2

Due to its potent antimicrobial activity the methanol extract (SCMEX: Table S3) was selected for further fractionation. SCMEX was dry loaded on to a 340 g Biotage SNAP ultra-cartridge attached to a Biotage Isolera Spektra System, and flash chromatography was performed with an ethyl acetate: methanol gradient (95%:5–5%5:95%). Fractions were dried and screened for antimicrobial activity against methicillin-resistant *S. aureus* (MRSA (ATCC 43300)) by a modified thin-layer chromatography bioautogram overlay method described elsewhere [31]. Bioautograms were developed by spraying with 3-(4,5-dimethylthiazol-2-yl) -2,5-diphenyltetrazolium bromide (MTT; Invitrogen, ThermoFisher Scientific, Paisley, UK) in PBS (0.5 mg/ml) and incubated for 30 min at 37 °C. Clear white/yellow zones against a purple background on the plate indicated the presence of antimicrobial compounds. Fractions 16–31 exhibited antimicrobial activity and were combined and loaded onto a Sephadex LH20 column (40 × 2.5 cm pre-equilibrated with 5%:95% (ddH20: MeOH)) and eluted with solutions of increasing MeOH concentration. 7 fractions (SCMBu1–7) each 20 ml in volume were obtained, but only fraction SCMBu5 retained antibacterial activity (Table S3). SCMBu5 underwent reversed-phase high performance chromatography (HPLC) using a W2690/5 HPLC system coupled with a W2996 photodiode array detector (Waters Corporation, Milford, USA) and using a Phenomenex Luna C18 (5u 100A 250 × 10 mm) column. Mobile phases A (water/acetonitrile 95%:5%) and B (acetonitrile/water 95%:5%) were prepared and a gradient elution programme (4.5 ml/min) of 0–5 min (98%; 2%), then 5–20 min (50%:50%) was performed. Two chromatographic peaks referred to as SC1 and SC2 were subsequently collected, dried, prepared at 16 mg/ml, filter sterilised and tested for antimicrobial activity. Molecular weights of SC1 and SC2 (Figs. S2 and S3) were determined using Q-TOF LC/MS (Waters Xevo G2-S Q-TOF system) (Column; Acquity UPLC HSS T3 column 100 Å, 1.8 μM, 2.1 mm × 100 mm) as previously described [32]. ESI parameters involved both negative and positive acquisition modes, a mass range 50-1200 Da, a desolvation gas temperature of 450 °C, desolvation gas flow of 14 L/min and the spray and cones voltages set at (1Kv and 30.0v, respectively). Structural verification studies involved Raman-IR spectroscopy (Thermo Scientific Nicolet iS5 FT-IR Spectrometer with Omnic Software™, Madison, Wisconsin, USA) and also ^13^C NMR and ^1^H NMR spectroscopy in CD_3_OD (400 MHz Bruker NMR, Billerica, MA, USA). The identities of SC1 and SC2 were confirmed by comparing chemical and spectroscopic properties reported against those in the literature.

### Effect of fractions in *galleria mellonella*

*G. mellonella* larvae (Livefoods Direct, Somerset, UK), were reared on an artificial diet at 25 °C (dark). During experiments, larvae were kept in an incubator at 37 °C in sterile Petri dishes (5 cm). Experimental groups consisted of 10 larvae (last instar) weighing 250–300 mg. In one series of experiments the ability of SCAF fractions to upregulate the number of haemocytes was determined. Sterile SCAF fractions of differing concentration were prepared in PBS and 20 μl aliquots were injected into the larvae hemocoel through proleg (left) in dorsolateral region using a Terumo Myjector 1 ml 29G (0.33 × 12 mm) needle [30]. Larvae were subsequently incubated for 24 h and haemolymph was removed, and haemocytes were enumerated using a haemocytometer. Secondly, the ability of pre-treatment of larvae with SCAF fractions to reduce bacterial load was investigated. Sterile SCAF0 or 5 fractions (20 μl of 100 μg/ml or PBS negative control) were injected as described above and after overnight incubation, larvae from each group were inoculated with 20 μl (2 × 10^3^ CFU MRSA; ATCC 43300) and maintained in an incubator at 37 °C. At 48 and 72 h post-infection, larvae (*n* = 7) from each group were removed and the bacterial load in haemolymph was determined by draining and diluting haemolymph. CFU/ml of haemolymph was determined by using the Miles and Misra Method to calculate CFU [31]. In another series of experiments, the question of whether *S. cordifolia* methanol fractions SC1 and 2 could improve resistance of *G. mellonella* to MRSA infection was evaluated. A bacterial suspension of MRSA (ACTCC 4330) was prepared and larvae were inoculated as described above. Six hours after infection, larvae were administered with either fraction (SC1 or SC2) and incubated (37 °C; 48 h), haemolymph was removed, and CFU/ml haemolymph were enumerated.

### Data analysis

Results are expressed as mean ± standard error (SEM). Differences in means were evaluated by one-way analysis of variance (ANOVA) with a post hoc Tukey’s test. Analyses were performed using GraphPad Prism 8.0 software (GraphPad, California, USA).

## Results

### Chemical and immunomodulatory properties of SCAF fractions

#### Chemical properties

The protein content of SCAF fractions was negligible indicating that polysaccharide was the major component. Expressed as a percentage, the dry weight protein content of SCAF0, 1, 2, 3, 4 and 5 were 0.8, 1.0, 2.1, 0.1, 0 and 0%, respectively. No endotoxin contamination was detected for any of the SCAF fractions even at lowest detection limit of the assay (0.03 EU/ml).

#### Immunomodulatory responses in splenocytes

**Fig. 1:**
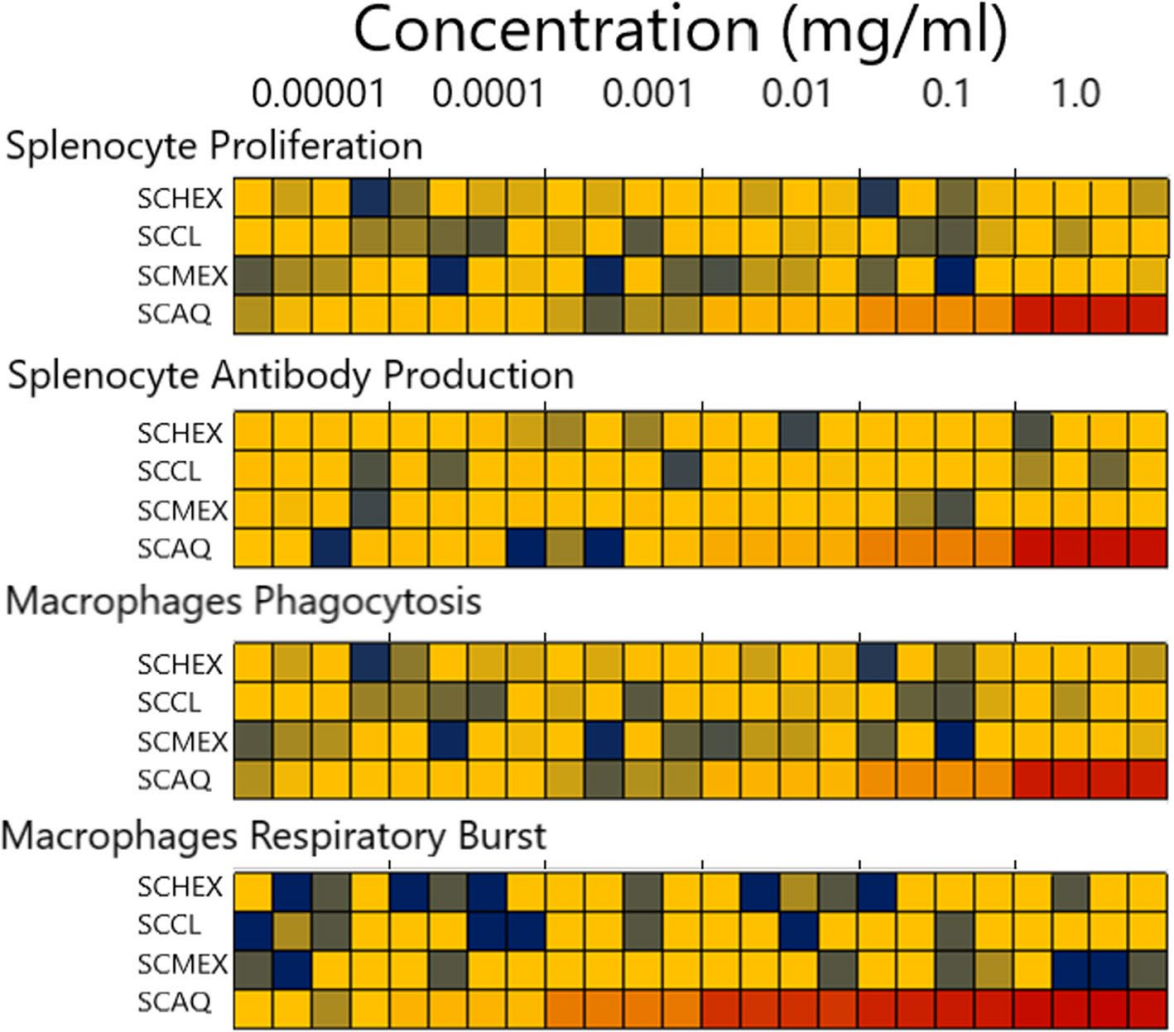
Screening of extracts for immunomodulatory activity. Crude extracts of *S. cordifolia* (SC) were prepared by sequential extraction using n-hexane (SCHEX), chloroform (SCCL), methanol (SCMEX) and water (SCAQ). Each crude extract was screened for immunomodulating activity using murine splenocyte proliferation (Alamar Blue assay), splenocyte antibody production, macrophage phagocytosis of neutral red and macrophage respiratory burst activity indirectly by measuring NO_2−_ production using Griess assay). Immunostimulatory activity in order from highest to lowest is red > yellow > blue

**Fig. 2:**
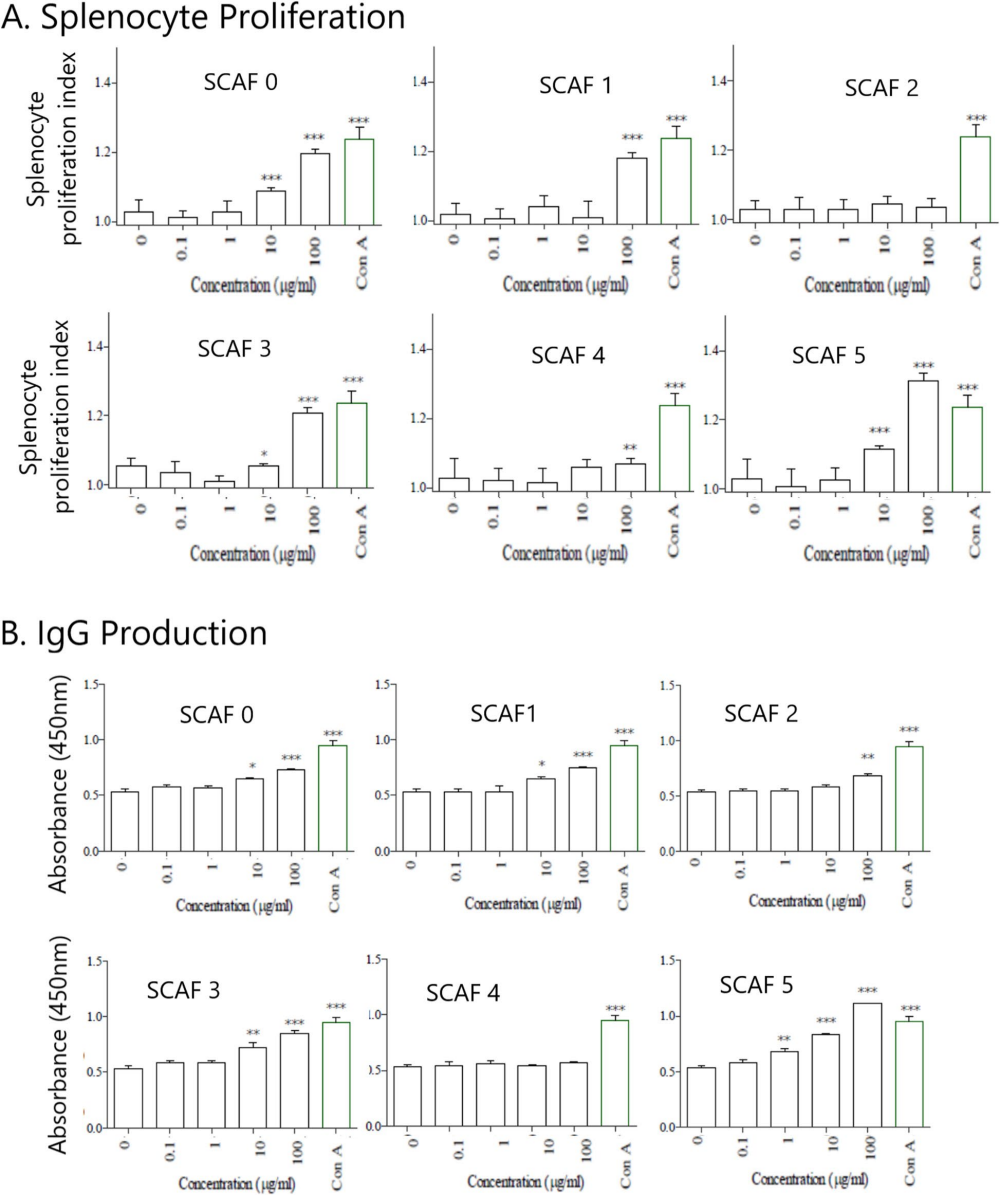
Immunomodulating activity of *S. cordifolia* ion exchange and size fractionated aqueous fractions (SCAF) on lymphocytes (splenocytes) in vitro. **A** Lymphoproliferation in response to SCAF0–5 treatment was measured using Alamar blue and results were expressed as proliferation index (OD treated cells divided by OD negative control cells). **B** Antibody secretion (IgG) following incubation with SCAF0–5 was measured by ELISA and results were expressed as optical density at 450 nm. Concanavalin A (Con A) was used as a positive control at a pre-determined optimal final concentration of 5 μg/ml and complete media was used as a negative non-proliferative control (0 μg/ml). All statistical analysis was carried out using a one-way ANOVA with Tukey’s Multiple Comparison Test to compare differences between samples and negative controls (*p < 0.05, **p < 0.01, ***p < 0.001, *n* = 3). Groups: SCAF 0 (crude polysaccharide fraction of *S. cordifolia*); SCAF 1: Low ionic strength < 100 kDa; SCAF 2: medium ionic strength and between 10 and 100 kDa; SCAF 3: medium ionic strength and > 100 kDa; SCAF 4 high ionic strength and between 10 kDa–100 kDa; SCAF 5: high ionic strength and < 100 kDa

The proliferative responses observed were moderate with the positive control (Con A) only eliciting a mean of 20.1% (+/− 1.4 standard deviation (SD)) increase in lymphoproliferation across all experiments. Of the initial extract fractions, only the aqueous extract (SCAQ) possessed immunomodulatory activity, significantly increasing lymphoproliferation and antibody production (Fig. 1). All SCAF fractions (except for SCAF4 (high ionic strength and between 10 and 100 kDa; *P* < 0.01)) elicited highly significant (*P* < 0.001) lymphoproliferative responses at the highest concentration (100 μg/ml) with a mean 13.2% (+/− 1.4 SD) increase observed across treatments compared with the negative control (Fig. 2A). SCAF5 induced the largest increase (27.3% +/− 0.83) which was greater in magnitude than the positive control (20.1%+/− 0.97). At 10 μg/ml, SCAF5 (8.4%+/− 0.15) followed by SCAF0 (5.9%+/− 0.11) induced the strongest response (*P* < 0.001). No response was detected to SCAF1, 2 and 4 at this concentration. Furthermore, no significant increase in lymphoproliferation was observed with any of the fractions at 1.0 μg/ml.

A more pronounced effect was observed with splenocyte antibody secretion (Fig. 2B) where SCAF5 elicited the greatest response by eliciting an approximate 2-fold increase (1.11 OD_450_+/− 0.08; 107.5% increase) compared to the negative control (0.54 OD_450_+/− 0.04) which was greater in magnitude than the positive control (0.98 OD_450_+/− 0.07; 83.5%). SCAF5 elicited statistically significant proliferative responses at 10.0 (*P* < 0.001) and 1.0 μg/ml (*P* < 0.01) but not at 0.1 μg/ml. Like the lymphoproliferative responses, all SCAF fractions elicited significant antibody production except for SCAF4.

#### Immunomodulatory responses in RAW 264.7 cells

**Fig. 3:**
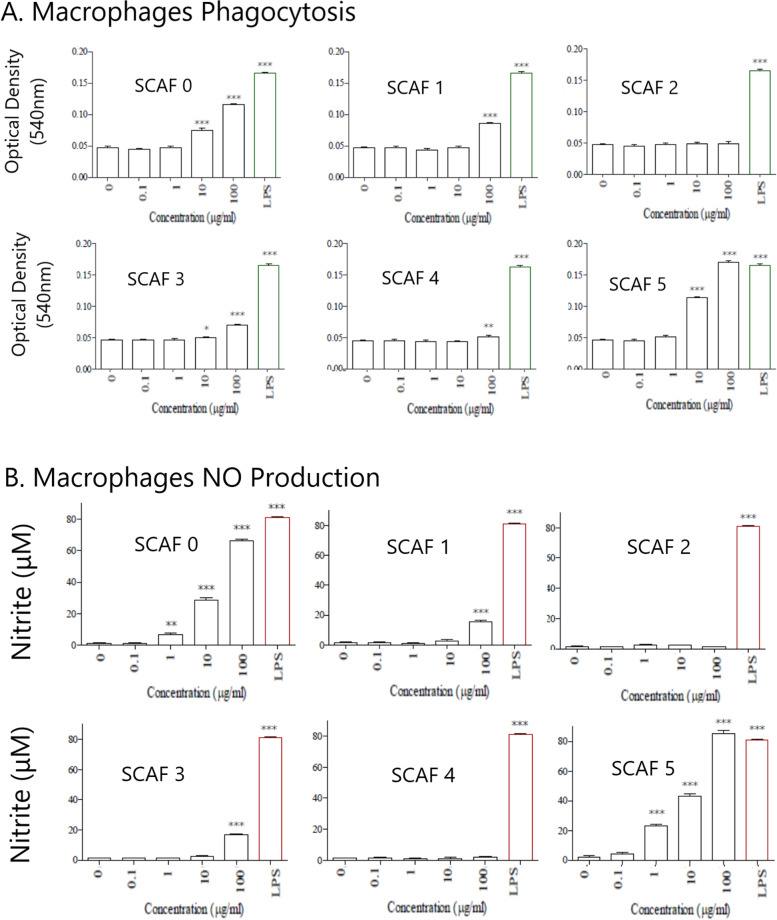
Immunomodulating activity of *S. cordifolia* ion exchange and size fractionated aqueous fractions (SCAF) on a macrophage cell line (RAW264.7) in vitro. **A** Macrophage phagocytic activity in response to SCAF0–5 treatment was measured using the Neutral Red phagocytosis assay and results were expressed as the optical density at 540 nm. **B** Production of nitric oxide indirectly by measuring NO_2−_ production in macrophages was achieved using the Griess reagent assay and expressed as μM. Lipopolysaccharide (LPS) was used a positive control at a pre-determined optimal concentration (1.0 μg/ml) and complete media was used as a negative control. All statistical analysis was carried out using a one-way ANOVA with Tukey’s Multiple Comparison Test to compare differences between samples and negative controls (*p < 0.05, **p < 0.01, ***p < 0.001, *n* = 3). Groups are SCAF 0 (crude polysaccharide fraction of *S. cordifolia*); SCAF 1: Low ionic strength < 100 kDa; SCAF 2: medium ionic strength and between 10 and 100 kDa; SCAF 3: medium ionic strength and > 100 kDa; SCAF 4 high ionic strength and between 10 kDa–100 kDa; SCAF 5: high ionic strength and < 100 kDa

Only the aqueous extract (SCAQ) significantly increased macrophage proliferation and nitric oxide (NO) production (Fig. 1). The largest increase in phagocytosis in SCAF-treated cells (Fig. 3A) was observed with 100 μg/ml SCAF5 (3.6-fold compared to negative control: *P* < 0.001) which was comparable to the LPS positive control (3.54-fold). This increase remained significantly different to the untreated cells at 10 μg/ml (2.4-fold; *P* < 0.001) but no phagocytic activity was observed at lower doses.

At 100 μg/ml, SCAF fraction 0 (66 μM+/− 2.1) and 5 (85 μM+/− 3.6) were the most potent stimulators of NO production (Fig. 3B) with the latter eliciting levels comparable to the positive control (81 μM+/− 3.2). SCAF5 also induced the strongest response at 10 μg/ml (43.3 μM+/− 2.5), at 1.0 μg/ml (23.3 μM+/− 1.5) and SCAF5 treatments from 100 to 1.0 μg/ml were highly significant (P < 0.001) compared to the negative control (1.1 μM+/− 0.01).

#### Cytokine expression

**Fig. 4:**
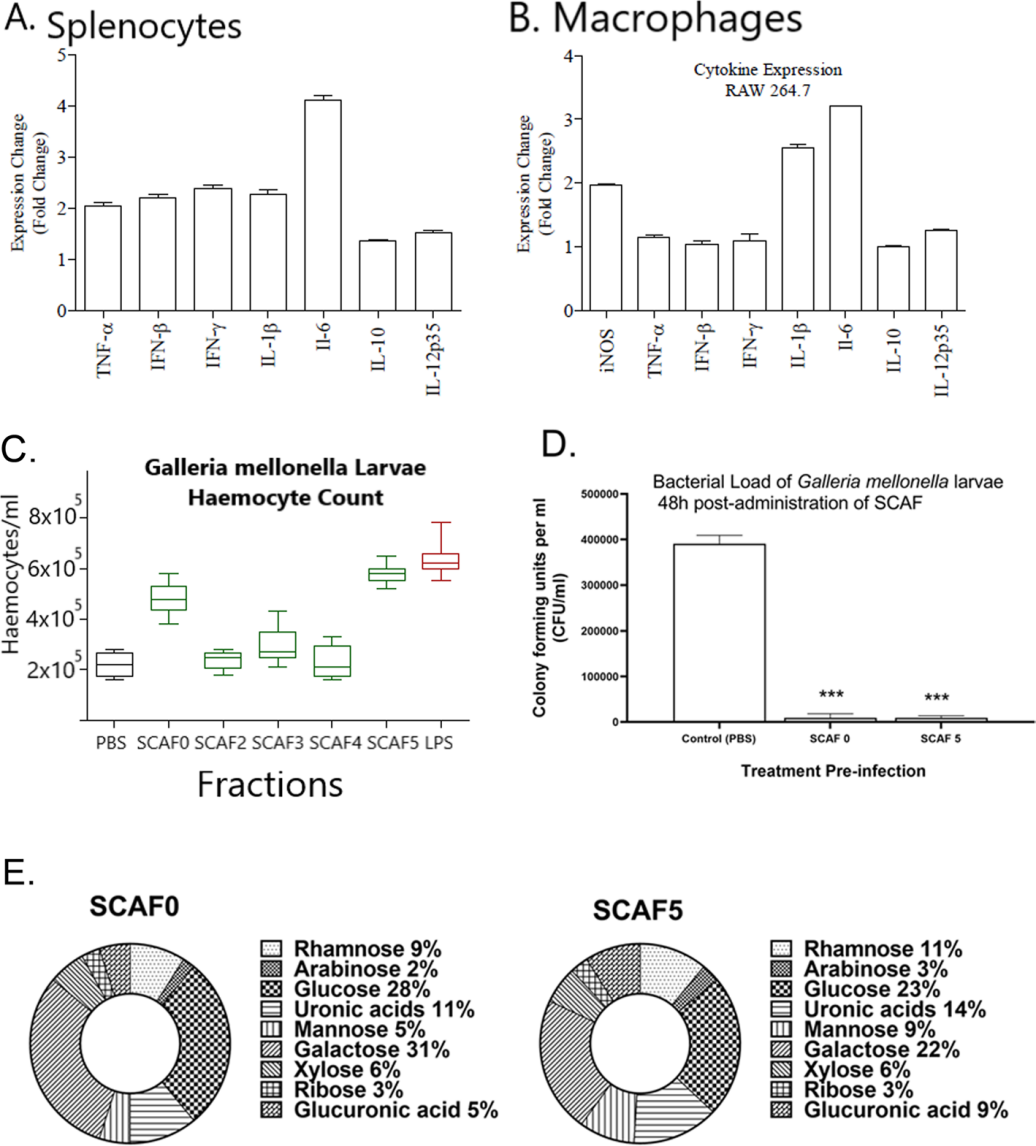
Activity and composition of *S. cordifolia* aqueous fractions (SCAF). Cytokine expression profiles in (**A**) lymphocytes (splenocytes) and (**B**) RAW 264.7 macrophage cell line following exposure to SCAF 5. (**C**) Effect of SCAF fractions on haemocyte counts (per ml of haemolymph) in *G. mellonella* larvae 24 h post-administration (SCAF0-SCAF5; 100 μg/ml, LPS 1 μg/ml). (**D**) Effect of pre-treatment of *G. mellonella* larvae with SCAF fractions on bacterial load. Larvae were inoculated with SCAF0 or 5 (20 μl of 100 μg/ml or PBS negative control) 24 h before infection with 20 μl (2 × 10^3^ CFU MRSA; ATCC 43300). At 48 h post-infection, larvae (*n* = 7) from each group were removed and the bacterial load (CFU/ml) in haemolymph was determined. (**E**) Monosaccharide composition of SCAF0 and SCAF5 as determined by GC-MS analysis (expressed as percentage of the total amount of monosaccharides present). All statistical analysis was carried out using a one-way ANOVA with Tukey’s Multiple Comparison Test to compare differences between samples and negative controls (*p < 0.05, **p < 0.01, ***p < 0.001, *n* = 8)

Cytokine gene expression in splenocytes and RAW 264.7 cells treated in vitro with SCAF5 as determined by RT-qPCR are shown in Figs. 4A and B. SCAF5 upregulated the expression of a wider range of proinflammatory cytokines in the mixed population of primary splenic lymphocytes (TNF-α, IFN-β, IFN-γ, and IL1-β and IL-6) than in the macrophages (IL1-β and IL-6). The most marked increase in SCAF5-induced cytokine expression levels was observed with IL-6 where a 4.2-fold increase (splenocytes) and 3.3-fold (macrophages) was observed compared with untreated controls. An approximate 2-fold increase in TNF-α, IFN-β, IFN-γ, and IL1-β was elicited in splenocytes whereas only IL1-β (~ 2.5-fold) was elevated to a similar magnitude in macrophages. SCAF5 also elicited a 2.1-fold increase in macrophage-associated iNOS expression in RAW264.7 cells which presumably leads to the increased NO generation. IL-10 and IL-12p35 levels were unaffected following SCAF5 treatment of either the splenic lymphocytes or macrophages.

#### In vivo immunomodulatory activity

Only SCAF0 (4.81 × 10^5^ haemocytes/ml) and SCAF5 (5.78 × 10^5^ haemocytes/ml) exhibited statistically significantly (*P* < 0.001) increases in haemocyte numbers in *Galleria* haemolymph compared to the negative control (2.2 × 10^5^ haemocytes/ml; Fig. 4C). SCAF5 exhibited the strongest increase compared to the unstimulated control in haemolymph proliferation (2.6-fold; SCAF0 = 2.2-fold increase) and its response was comparable to the LPS positive control (2.9-fold; 6.33 × 10^5^ haemocytes/ml). The antimicrobial effect of this immunostimulatory activity was determined by measuring bacterial load in larvae pre-treated with either SCAF0 or 5. Bacterial load in haemolymph from larvae (*n* = 7) pre-treated with PBS (negative control) was 3.31 × 10^5^ CFU/ml 48 h post-infection with MRSA (ATCC 43300) (Fig. 4D). Pre-treatment with either SCAF0 (8.57 × 10^3^ CFU/ml) or 5 (9.29 × 10^3^ CFU/ml) resulted in an extremely statistically significant (*P* < 0.001) 98% drop in bacterial load compared to the negative control with 2/7 larvae exhibiting zero bacterial load. This reduction in load was maintained to the end of the study (72 h post-infection) in the SCAF0 (4.29 × 10^3^ CFU/ml) and 5 fraction (2.86 × 10^3^ CFU/ml) with 3/7 larvae in both groups exhibiting zero bacterial load (data not shown).

#### Monosaccharide composition

The monosaccharide composition of polysaccharides contained in SCAF0 and SCAF5 is outlined in Fig. 4E. Both fractions were composed of at least 9 different monosaccharides, but it would be expected that the monosaccharide profile of SCAF5 more accurately reflects the composition of the bioactive polysaccharides than that of SCAF0. SCAF5 polysaccharides possessed approximately 1.8-fold more glucuronic acid and mannose, and ~ 1.3-fold more uronic acid. Conversely, it contained less galactose (1.4-fold) and glucose (0.8-fold), with the content of other monosaccharides not differing.

### Chemical and antibacterial properties of SC fractions

#### Chemical properties of SC1 and SC2

**Fig. 5:**
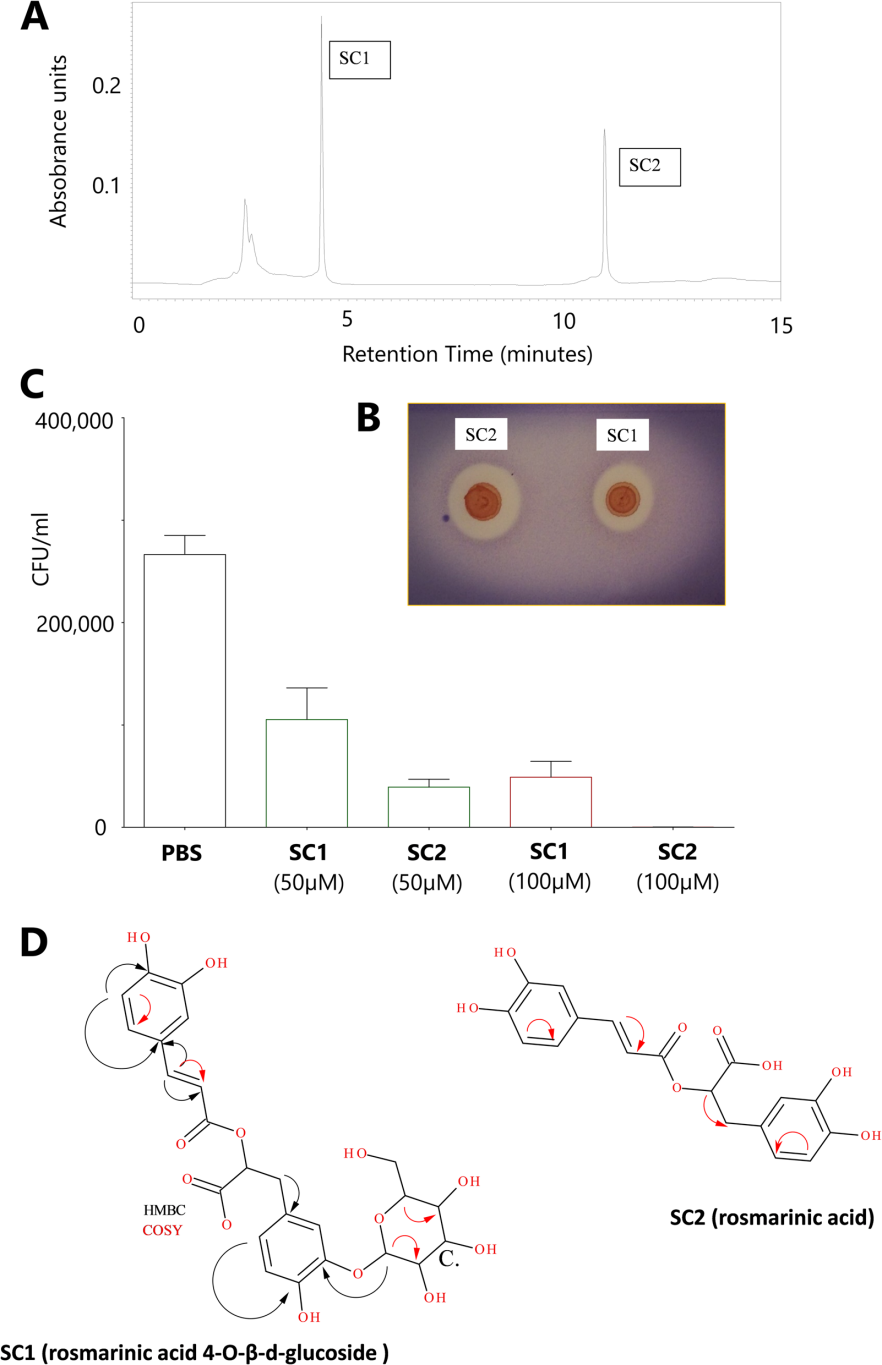
Antimicrobial compounds isolated from *S. cordifolia.* (**A**) Chromatogram showing HPLC separation of fraction SCMEXBu5. Two peaks designated as SC1 and SC2 were both isolated, both of which exhibited antibacterial activity (**B**) An example of a bioautography representing in vitro antimicrobial activity. Zones of inhibition were produced by spotting SC1 or SC2 (50 μg) on a TLC overlaid with Mueller-Hinton agar inoculated with MRSA 4330. The resulting zones were visualised by spraying with MTT dye (**C**) In vivo antimicrobial activity of SC1 and SC2 against MRSA ATCC 43300-infected *Galleria* larvae (**D**) 2D COSY and HMBC experiments were highly suggestive of the proposed structure of SC1 representing rosmarinic acid 4-O-β-d-glucoside and SC2 representing rosmarinic acid

SC1 and SC2 were isolated as orange amorphous powders. The calculated molecular formula for SC2 was C_18_H_16_O_8_; ESI- MS (negative ion) *m/z* 359.0773 [M-H] (Figs. S2) and for SC1 was C_24_H_26_O_13_; ESI- MS (negative ion) *m/z* 521.1302 (Fig. S3). The UV λ max for both SC1 and SC2 were 222, 286 and 328 nm suggestive of a phenolic chromophore (Fig. 5A). IR spectra for both SC1 and SC2 were similar for both compounds IRνmax O-H 3334 cm^− 1^, C=C (aromatic) 1600–1475 cm^− 1^, C-O 1320–1210 cm^− 1^, C=O 1696–1721 cm^− 1^ (data not shown). The ^1^H NMR spectrum of SC2 (Fig. S2) showed two doublets at δ7.44 (7a) and δ6.34 (8a) which based on the large H-H coupling (J15.8) were assigned to the trans-olefinic protons 7a and 8a. 2D NMR ^1^H-^1^H (COSY) coupling were suggestive of coupling between 5a (δ 6.62) - 6a (δ 6.52) and 5b (δ 6.77) - 6b (δ 6.85) on the 3, 4-dihydroxyphenyl units. ^13^C NMR spectra of SC2 showed the presence of two carbonyl carbons of which one was attributed to a carboxylic carbon (δ173.51) and the other to a carboxyl ester δ168.24. The presence of two sets of 3,4-dihydydrophenyl groups was confirmed by the presence of 12 aromatic carbons, six were quaternary carbons of which 4 were identified as phenoxyl carbons (δ146–151) (3a, δ 147.64, δ 149.723b δ146.16 and 4b δ 145.26) in addition to the two olefinic carbons identified 7a δ 146.16 and 8a δ 117.59, 2D NMR ^1^H -^13^C (HMBC) indicated these carbons were coupled to transolefinic protons, SC2 was subsequently identified as rosmarinic acid (Figs. S2 and Fig. 5D).

SC1 exhibited a very similar NMR profile to SC2 suggesting the presence of rosmarinic acid (Fig. S3), 6 additional carbons were observed corresponding to a sugar (δ102.52, 73.02, 73.06, 40.95, 74.95 and 64.5), carbon 1c (δ 102.52) which identified as being linked to 3b by long range coupling SC1 was subsequently identified as rosmarinic acid 4-O-β-d-glucoside (Rosmarinyl glucoside). Mass fragmentation patterns obtained for both SC1 (rosmarinic acid 4-O-β-d-glucoside) and SC2 (rosmarinic acid) were consistent with the proposed structures (Fig. 5D).

#### In vitro antibacterial activity of SC1 and SC2

The initial methanol extract possessed no antibacterial activity against Gram-negative bacteria *E. coli* (ATCC 11303) and *P. aeruginosa* (PAO1) but exhibited bacteriostatic activity against Gram-positive organisms with MIC values ranging from 0.5–2.0 mg/ml (Table 1). The intermediate fraction SCMEXBu5 exhibited bacteriostatic and bactericidal activity against the Gram-positive bacteria *Staphylococcus aureus* (SA), *Enterococcus faecalis* (EF), *Pseudomonas aeruginosa (PAO1),* Methicillin-Resistant *Staphylococcus aureus* (MRSA) and Methicillin-Resistant *Staphylococcus epidermidis* (MRSE) (Table S3). SCMEXBu5 exhibited no activity against Gram-negative organisms (data not shown). After further fraction and purification by HPLC two peaks with identical UV λ max profiles (Fig. 5A) were isolated (SC1 & SC2). SC1 and SC2 both exhibited similar antibacterial potency (MIC and MBC) against an array of Gram-positive bacteria (Table 2). Both compounds exhibited the greatest potency against the antibiotic-resistant MRSA strains (1.25 mmol MIC/MBC) and to a lesser extent against MRSE (5.0–20 mmol MIC/MBC). SC1 and SC2 possessed no bioactivity against Gram-negative microorganisms (data not shown).

**Table 1:** Antimicrobial activity of *S. cordifolia* extracts

	MRSA ACTCC 43300	SA ATCC 35984	MRSE NCTC11964	PAO1	E.coli ATCC 11303
**SCHEX**	> 4	> 4	> 4	> 4	> 4
**SCCL**	> 4	> 4	> 4	> 4	> 4
**SCMEX**	0.5	1	2	> 4	> 4
**SCAQ**	4	> 4	> 4	> 4	> 4

Minimum inhibitory concentrations (MIC) expressed as mg/ml of crude hexane (SCHEX), chloroform (SCCL), methanol (SCMEX) and aqueous (SCAQ) extracts of *S. cordifolia*. The crude extracts were prepared as a 2X working stock (16 mg/ml), filter sterilised and screened using a concentration range from 8000 to 0.003 μg/ml. MRSA: Methicillin-Resistant *Staphylococcus aureus.* SA: *Staphylococcus aureus,* MRSE Methicillin-Resistant *Staphylococcus epidermidis*, PAO1: *Pseudomonas aeruginosa and E. coli*: *Escherichia coli*

**Table 2:** Antimicrobial activity of compounds isolated from *S. cordifolia*

	MRSA	MRSA	SA	SA	SE	MRSE	SE	EF
	ATCC 43300	ATCC 33591	NCTC 12493	ATCC 29213	ATCC 35984	NCTC 11964	ATCC 12228	DZMZ 25390
***Mean Inhibitory Concentration (MIC) (mmol)***								
**SC 1**	2.5	2.5	5	2.5	20	10	20	20
**SC 2**	1.25	1.25	2.5	2.5	20	5	10	10
***Mean Bactericidal Concentration (MBC) (mmol)***								
**SC 1**	2.5	2.5	5	5	20	20	40	40
**SC 2**	1.25	1.25	5	5	20	10	20	20

Minimum inhibitory concentrations (MIC) and mean bactericidal concentrations (MBC) of *S. cordifolia* fractions SC1 and SC2 (expressed as mmol). MRSA: Methicillin-Resistant *Staphylococcus aureus*, MRSE Methicillin-Resistant *Staphylococcus epidermidis*, SA: *Staphylococcus aureus*, EF: *Enterococcus faecalis* and SE *Staphylococcus epidermidis*

#### In vivo antibacterial activity of SC1 and SC2

Bioautography by means of agar overlay visibly showed the in vitro antibacterial activities of SC1 and SC2 against MRSA (ATCC 43300; Fig. 5B). The in vivo antibacterial activity against this antibiotic-resistant strain was analysed by injecting *Galleria* larvae with either SC1 or SC2 (50 or 100 μM dose in PBS) 24 h before challenge with MRSA (Fig. 5C). Injection of larvae with SC2 (100 μM) 24 h prior to MRSA challenge led to a markedly lower bacterial load (1243-fold decrease; 2.1 × 10^2^ CFU/ml) compared to the PBS negative control (2.66 × 10^5^ CFU/ml). In fact, bacterial growth of the antibiotic-resistant strain was almost completely abrogated with no bacteria cultivated from 5 of 7 larvae. The antibacterial activity of SC2 decreased at doses lower than 100 μM (50 μM; 6.8-fold decrease compared to negative control: 3.94 × 10^4^ CFU/ml). Treatment with 100 μM and 50 μM of SC1 caused a 5.4-fold (4.9 × 10^4^ CFU/ml) and 2.5-fold (1.05 × 10^5^ CFU/ml) decrease in bacteria counts, respectively, compared to the negative control.

## Discussion

The dual immunomodulatory and antimicrobial activity of *S. cordifolia* revealed by this study indicates its potential usefulness for treating and/or preventing bacterial infection. Such properties could be developed as (i) an alternative approach to clinically proscribed antibiotics, but equally, (ii) a ground root extract could be developed as a feed additive for the agri-food industry. This would result in harnessing the ability of polysaccharides to non-specifically stimulate the immune system in combination with the well documented ability of rosmarinic acid to inhibit bacterial growth. The replacement of antibiotics in livestock could slow, and potentially reverse, the emergence of antibiotic resistance in a matter of years as has been demonstrated by the ban of vancomycin antibiotics in the Netherlands [33]. Companion animals are also an emerging source of concern in the field of antibiotic resistance, with several microbiological threats from pets, including MRSA, having been identified as potential threats to humans [9, 10].

Our initial in vitro screening of crude root extracts (see Fig. 1) found only the aqueous fraction (SCAQ) possessed immunomodulating activity as measured by lymphocyte stimulation (cell proliferation and increased antibody secretion) and macrophages activation (enhanced phagocytosis and NO production). Ethanol precipitation of the aqueous extraction resulted in the isolation of pale-brown precipitate (SCAF0) also with immunomodulatory activity. Biochemical analysis (Molisch’s test; data not shown) indicated the presence of carbohydrates. Further fractionation (based on ionic strength and molecular size filtration) of this carbohydrate-enriched extract produced five fractions (SCAF1–5). A further round of in vitro immunological screening identified that the fraction with highest ionic strength and largest molecular size (> 100 kDa), SCAF5, had the most potent immunomodulatory activity. SCAF5 (100 μg/ml) elicited potent lymphoproliferative activity equivalent to the positive control lectin mitogen, and the bioactivity of this fraction was further confirmed by the marked increases in antibody levels in treated splenocytes as well as macrophage activation. Given that protein and potential immunomodulating LPS contaminants were undetectable in the bioactive fraction, the immunomodulatory activity shown here can be attributed solely to plant polysaccharides. Hydrolysis and GC-MS analysis determined the monosaccharide composition of polysaccharides present in SCAF0 and SCAF5. The profiles obtained demonstrate that these fractions remain impure and contain a complex mixture of different polysaccharides. It is likely that key components of the plant cell wall, such as rhamnogalacturonan-I (RG-I), homogalacturonan (HG), xylogalacturonan (XGA), rhamnogalacturonan-II (RG-II) and other polysaccharides are present and each of these could potentially contribute to the overall immunomodulatory effects observed. Indeed, it has been demonstrated elsewhere that some of these polysaccharide structures from certain plant roots have immunological activity [34]. For example, a high-molecular weight arabinogalactan branched RG-I, isolated from the roots of *Vernonia kotschyana*, has shown T-cell-independent induction of B cell proliferation and induces the chemotaxis of human macrophages, T-cells, and natural killer (NK) cells [35]. Similarly, arabinogalactan-containing RG-I domains from hot water solublised pectic fractions from the roots of *Panax ginseng* were effective in causing lymphocyte proliferation [9]. Much work remains to be done to understand the specific motifs within these complex cell wall polysaccharides responsible for these observed immunological properties. Since the immunomodulatory effects observed here are quite diverse it is unclear if these are the results of a single polysaccharide, or if multiple polysaccharides are acting in synergy. To elucidate this, it will be necessary to fragment, purify, structurally characterise and test individual molecules from within the SCAF5 fraction. The reason why only certain plant species are rich in immunological activity is also unclear, but an improved understanding of the precise polysaccharide structures involved should also help elucidate this.

Proinflammatory cytokines (e.g. IFN-γ and TNF_-α_) are produced by macrophages which increase expression of inducible NO synthase (iNOS), resulting in enhanced NO production [36]. SCAF5 stimulated NO production in macrophages, and these results were corroborated by RT-qPCR analysis which showed a two-fold increase in iNOS mRNA expression (Fig. 4). Increases in the expression of iNOS and SCAF5-induced NO observed in this study are indicative of a T_H_1-mediated response [37]. There are several T_H_ cell subsets classified based on their unique cytokine profiles and functions [38],with the T_H_1-mediated response contributing towards microbicidal activity (i.e., NO production and phagocytosis), which are highly effective in clearing intracellular pathogens (e.g., bacteria and viruses) compared with the T_H_2 phenotype which is implicated in less desirable hypersensitivity reactions [39]. Further evidence for the activation of a T_H_1 microbicidal response was observed with the 2-fold increase in transcripts for T_H_1-associated cytokines interferon (IFN-γ) and tumour necrosis factor (TNF-α). A further indication that SCAF5 promotes an antimicrobial T_H_1-mediated immune response is evidenced by a lack of expression of the T_H_2-associated cytokine IL-10 in both cell types. The most marked increase in cytokine expression in macrophages and splenocytes was that of IL-6. This pro-inflammatory cytokine is secreted by T cells and activated macrophages following activation by microbial PAMPs and is critical for defence against a number of intracellular pathogens [40]. IL-6 induces antibody production and activation of macrophages and T-cells which corresponds to our experimental observations. An approximate doubling in the levels of expression of IL-1β (splenocytes and macrophages) and IFN-β (splenocytes) was also observed with SCAF5.

In vivo immunomodulation was confirmed in a novel model of innate immunity using the insect larvae from *Galleria mellonella (G. mellonella*)*.* These larvae are an inexpensive and ethically favourable alternative to animal anti-microbial infection models due to its susceptibility to infection, larger size facilitating experimental manipulation, short life cycle and ability to grow at physiological temperatures (37 °C) [21, 41]. Intriguingly insects possess a highly effective immune system containing humoral and cellular components with many similarities to the vertebrate innate immune system. For example, their cellular component is composed of phagocytes (haemocytes) which possess the capacity to detect highly conserved antimicrobial pathogen associated molecular patterns (PAMPs) [42]. The haemocytes of *G. mellonella* are analogous to vertebrate immune cells such as macrophages because they are capable of phagocytosis, and they express pathogen recognition receptors (PRR) for detecting pathogens. The immunomodulatory activity of SCAF5 observed in vitro was reproduced in vivo following administration of either SCAF fraction (SCAF0 or 5) in this primitive arthropod model, and SCAF5 was again found to exhibit the most potent activity as determined by a marked increase in the number of haemocytes in haemolymph. The proliferation of haemocytes was almost equivalent in magnitude to the response elicited by the pre-optimised LPS positive control. Subsequently larvae pre-treated with SCAF5 were extremely resistant (*P* < 0.001) to infection with methicillin-resistant *Staphylococcus aureus* (MRSA) with a 98% reduction in bacterial load compared to the negative control.

Ion exchange and size fractionation indicates that SCAF5 possesses a high ionic strength and a molecular size greater than 100 kDa, which was confirmed by electrophoretic studies which indicated the size of the bioactive component within this fraction ranged from 100 kDa–150 kDa (data not shown). SCAF2 and SCAF4 with molecular weights ranging from 10 to 100 kDa exhibited more moderate activity profiles and suggesting that the size of the bioactive component may be an important determinant for the immunomodulatory activity. GC-MS analysis of SCAF5 confirmed the bioactive component consisted of polysaccharides and that the type of monomer constituents were similar, but that the ratio of sugars differed. SCAF5 contained higher levels of both mannose and glucuronic and uronic acid and this may explain the need for a high ionic strength solution to elute SCAF5. The increased proportion of mannose in SCAF5 may suggest the immunomodulatory activity is mediated via the mannose PRR found on immune cells [43]. Alternatively, glucuronic acid is a functional group of the commercial adjuvant QS-21 which essential for its immunogenic function [44].

Preliminary in vitro antimicrobial susceptibility screening assays indicated that the crude methanol fraction contained compounds of interest. Fractionation by flash chromatography resulted in the isolation of the bioactive active fraction SCMEXBu5 and subsequent HPLC fractionation elucidated two peaks (SC1 & SC2) with identical UV λ max profiles. Bioautography by means of agar overlay confirmed both SC1 and SC2 exhibited antibacterial activities against methicillin-resistant *S. aureus* (MRSA) and the source of the antimicrobial activity was subsequently identified as rosmarinic acid 4-O-β-d-glucoside (SC1) and rosmarinic acid (SC2). The activity was specific to Gram-positive bacteria including antibiotic resistant strains of *Staphylococcus aureus* and *Staphylococcus epidermidis* and the observed selectivity for Gram-positive bacteria suggests the mechanism of action involves targeting the cell wall. The bactericidal and bacteriostatic activity of SC2 reported in this study are equivalent in concentration range (mg/ml) as other antimicrobial agents tested against the same panel of reference strains [27].

The antimicrobial properties of rosmarinic acid and its derivatives has been described previously [8] and these studies also confirm our observed specificity for Gram-positive bacteria. No mechanism of action has yet been elucidated although it has been suggested that rosmarinic acid targets cell surface virulence factors unique to Gram-positive bacteria which mediate the initial host-bacteria interactions [45, 46]. Interestingly, both in vitro and in vivo studies have provided extensive evidence that rosmarinic acid is a potent and effective anti-tumour agent [47–49] and studies are currently underway to elucidate its putative anti-cancer properties with crude and fractionated *S. cordifolia* extracts. Antimicrobial activity was demonstrated to also be effective in reducing in vivo microbial load in *Galleria* larvae which were infected with MRSA. Subsequent administration of the highest concentration of SC2 (100 μM) decreased in vivo MRSA load by 99.9% (a 1243-fold decrease in CFU/ml) in haemolymph and a complete elimination of bacteria was observed in 5 out of 7 larvae.

## Conclusion

*S. cordifolia* exhibits dual antimicrobial activities in that it stimulates the immune system via a T_H_1-mediated antimicrobial response, priming a host in readiness for a potential infection as demonstrated with the *G. mellonella* in vivo model. The presence of compounds with direct antimicrobial activity (against Gram-positive bacteria including MRSA) is effective in limiting the extent of an infection in a host already infected. A more detailed characterisation of the polysaccharides involved will enable progress to be made in respect to fully understanding the properties of this extract and its potential applications. Nonetheless, the fact that rosmarinic acid is also reported to have antiviral activity indicates that there is potential for using *S. cordifolia* crude extracts as an inexpensive animal feed supplement to prevent infectious disease and reduce the use of antibiotic growth promoters in livestock and companion animals.

## Supplementary Information

**Additional file 1 Table S1.** Composition of *S. cordifolia* aqueous fractions (SCAF). **Table S2.** Primers sequences (forward and reverse) used for quantification of cytokine mRNA expression in splenocytes and RAW246.7 macrophage cell line. **Table S3:** MIC and MBC (mg/ml) of Sephadex LH-20 fractions (SCMEXBu) compared to crude methanol extract (MEX). **Fig. S1.** (A) Typical melt curve for murine inducible nitric oxide synthase (iNOS) (B) Amplification curve of iNOS following incubation of RAW 264.7 cells with SCAF 5 showing untreated control versus treatment with SCAF 5. (C) Amplification for IL-6 following incubation of splenocytes with SCAF 5. Lines show untreated control versus treatment with SCAF 5. **Fig. S2.** Mass Spectrum of SC2 (A) Mass Fragmentation patterns obtained for SC2 were suggestive of rosmarinic acid. (B) ^13^C NMR spectra of SC2. (C) Table summarising chemical shifts of both ^13^C NMR and ^1^H NMR spectra for SC2. ^13^C NMR and ^1^H NMR shifts and proposed structure of SC2 were highly suggestive of rosmarinic acid. (D) The proposed structure of SC2 (rosmarinic acid). **Fig. S3.** Mass Spectrum of SC1 (A) Mass Fragmentation patterns obtained for SC1 were suggestive of rosmarinic acid 4-O-β-d-glucoside (Rosmarinyl glucoside). (B) ^13^C NMR spectra of SC1. (C) Table summarising chemical shifts of both ^13^C NMR, and ^1^H NMR. ^13^C NMR, and ^1^H NMR shifts were highly suggestive of rosmarinic acid 4-O-β-d-glucoside. and the (D) proposed structure of SC1 (rosmarinic acid 4-O-β-d-glucoside).

## Data Availability

The data used to support the findings of this study are included within the article and any further information can be provided by corresponding author upon request.

## Abbreviations

Abs. Ethanol: Absolute Ethanol
ATCC: American Type Tissue Culture
BRM: Biological Response Modifiers
BSTFA: N,O-Bis (trimethylsilyl)trifluoroacetamide
cDNA: Complementary deoxyribonucleic acid
CD3OD: Deuterated Methanol
CFU: Colony forming units
CLSI: Clinical and Laboratory Standards Institute
Con A: Concanavalin A
COSY: Correlation Spectroscopy
Ct: Cycle threshold
ddH2O: Double Distilled Water
DMEM: Dulbecco’s Modified Eagle’s Medium
*E. coli*: *Escherichia coli*
ECACC: European Collection of Cell Cultures
EF: *Enterococcus faecalis*
ELISA: Enzyme-linked Immunosorbent Assay
ESI: Election Spray Ionisation
EtOH: Ethanol
EU: Endotoxin unit
EXAP: Polysaccharide Enriched Fractions (Polysaccharide Precipitate from Aqueous Extract)
FBS: Foetal Bovine Sera (Heat Inactivated)
FT-IR: Fourier Transform- Infra-Red Spectroscopy
GC-MS: Gas Chromatography – Mass Spectroscopy
H2SO4: Hydrosulphuric acid
HPLC: High Performance Liquid Chromatography
HPRT1: Hypoxanthine Guanine Phosphoribosyl Transferase 1
HRP: Horseradish Peroxidase
IFN: Interferon
IgG: Immunoglobulin G
IL: Interleukin
iNOS: Inducible Nitric Oxide Synthase
LAL: Limulus Amebocyte Lysate
LPS: Lipopolysaccharide
MBC: Minimum Bactericidal Concentration
MeOH: Methanol
MHA: Müller-Hinton Agar
MHB: Müller-Hinton Broth
MIC: Minimum Inhibitory Concentration
MIQE: Minimum Information for Publication of Quantitative Real-Time PCR Experiments
MRSA: Methicillin Resistant *Staphylococcus aureus*
MRSE: Methicillin Resistant *Staphylococcus aureus*
MS: Mass Spectroscopy
MTT: 3-(4,5-dimethylthiazol-2-yl) -2,5-diphenyltetrazolium bromide
MWCO: Vivaspin™ Molecular Cut-Off Filters
NCTC: National Collection of Type Cultures
NIST: National Institute of Standards and Technology
NMR: Nuclear Magnetic Resonance
NO: Nitric Oxide
NOS: Nitric Oxide Synthase
NR: Neutral Red
OD: Optical Density
PAMP: Pathogen-Associated Molecular Patterns
PAO1: *Pseudomonas aeruginosa*
PBS: Phosphate Buffered Solutions
PI: Proliferation Index
PRR: Pattern Recognition Receptor
RPMI: Roswell Park Memorial Institute
RNA: Ribonucleic acid
SA: *Staphylococcus aureus*
SE: *Staphylococcus epidermidis*
SC: Methanol
SCAF: SCAP size/ionic strength fractionated
SQAP: *Sida Cordifolia* crude aqueous portion
SCCL: *Sida Cordifolia* crude chloroform portion
SCHEX: *Sida Cordifolia* crude hexane portion
SCMEX: *Sida Cordifolia* crude methanol portion
SCMEXBu: *Sida Cordifolia*
SPE: Solid Phase Extraction
TFA: Trifluoroacetic acid
TLC: Thin Layer Chromatography
TLR: Toll-Like Receptor
TMB: 3,3, 5,5′ Tetramethyl Benzidine
TNF: Tumour Necrosis Factor
WHO: World Health Organisation
