# Supplementary material for: Extracts of Sida cordifolia contain polysaccharides possessing immunomodulatory activity and rosmarinic acid compounds with antibacterial activity
Source: BMC Complement Med Ther. 2022 Jan 27;22:27. doi: 10.1186/s12906-022-03502-7 (PMC8793188; doi:10.1186/s12906-022-03502-7)
Supplement: Supplementary file 1 — Additional file 1 Table S1. Composition of S. cordifolia aqueous fractions (SCAF). Table S2. Primers sequences (forward and reverse) used for quantification of cytokine mRNA expression in splenocytes and RAW246.7 macrophage cell line. Table S3: MIC and MBC (mg/ml) of Sephadex LH-20 fractions (SCMEXBu) compared to crude methanol extract (MEX). Fig. S1. (A) Typical melt curve for murine inducible nitric oxide synthase (iNOS) (B) Amplification curve of iNOS following incubation of RAW 264.7 cells with SCAF 5 showing untreated control versus treatment with SCAF 5. (C) Amplification for IL-6 following incubation of splenocytes with SCAF 5. Lines show untreated control versus treatment with SCAF 5. Fig. S2. Mass Spectrum of SC2 (A) Mass Fragmentation patterns obtained for SC2 were suggestive of rosmarinic acid. (B) 13C NMR spectra of SC2. (C) Table summarising chemical shifts of both 13C NMR and 1H NMR spectra for SC2. 13C NMR and 1H NMR shifts and proposed structure of SC2 were highly suggestive of rosmarinic acid. (D) The proposed structure of SC2 (rosmarinic acid). Fig. S3. Mass Spectrum of SC1 (A) Mass Fragmentation patterns obtained for SC1 were suggestive of rosmarinic acid 4-O-β-d-glucoside (Rosmarinyl glucoside). (B) 13C NMR spectra of SC1. (C) Table summarising chemical shifts of both 13C NMR, and 1H NMR. 13C NMR, and 1H NMR shifts were highly suggestive of rosmarinic acid 4-O-β-d-glucoside. and the (D) proposed structure of SC1 (rosmarinic acid 4-O-β-d-glucoside). [file 12906_2022_3502_MOESM1_ESM.docx]

**Supplementary Data**

**Table S1.** Composition of *S. cordifolia* aqueous fractions (SCAF).

| **Fractions** | **Fraction composition (dry weight)** |
| --- | --- |
| SCAF0 | crude polysaccharide fraction of *S. cordifolia* (SCAQ) |
| SCAF1 | low ionic strength and <100kDa |
| SCAF2 | medium ionic strength and between 10-100kDa |
| SCAF 3 | medium ionic strength and < 100kDa |
| SCAF 4 | high ionic strength (2 mol/l) and between 10kDa-100kDa |
| SCAF 5 | high ionic strength and <100kDa |

The crude aqueous extract (SCAQ) was precipitated using ethanol (EXAP) and then further fractionated on the basis of ionic strength and size.

**Table S2.** Primers sequences (forward and reverse) used for quantification of cytokine mRNA expression in splenocytes and RAW246.7 macrophage cell line.

| Gene | Forward | Reverse | Reference |
| --- | --- | --- | --- |
| IL-1a | GGAAGATTCTGAAGAAGAGACGG | TGAGATTTTTAGAGTAACAGG | (67) |
| IL-1b | TGTCTGAAGCAGCTATGGCAAC | CTGCCTGAAGCTCTTGTTGATG | (68) |
| IL-6 | TCTTGGGACTGATGCTGGTG | CAGAATTGCCATTGCACAACTC | (68) |
| IL-10 | AATTCCCTGGGTGAGAAGCTG | CATGGCCTTGTAGACACCTTG | (68) |
| 1L-12 p35 | TGG ACC TGC CAG GTG TCT TAG | CAATGTGCTGGTTTGGTCCC | (69) |
| IL-12 p40 | AAGAAGGAAAATGGAATTTGGTCC | ATGTCACTGCCCGAGAGTCAG | (69) |
| TNF - α | CTCAGCCTCTTCTCATTCCTGC | CCATAGAACTG ATGAGAGGG | (68) |
| IFN-γ | AGCAACAGC AAG GCG AAA A | CTGGACCTGTGGGTTGTTGA | (67) |
| IFN-β | CGTGGGAGATGTCCTCAACT | AAGATCTCTGCTCGGACCAC | (67) |
| iNOS | CAG CTG GGC TGT ACA AAC CTT | CAT TGG AAG TGA AGC GTT TCG | (69) |
| HPRT1 | GAGGAGTCCTGTTGATGTTGCCAG | GGCTGGCCTATAGGCTCATAGTGC | (70) |

HPRT1 was used as the reference gene for the normalisation of all RT-qPCR assays.

**Figure S1.** (A) Typical melt curve for murine inducible nitric oxide synthase (iNOS) (B) Amplification curve of iNOS following incubation of RAW 264.7 cells with SCAF 5 showing untreated control versus treatment with SCAF 5. (C) Amplification for IL-6 following incubation of splenocytes with SCAF 5. Lines show untreated control versus treatment with SCAF 5.

**Figure S2.** Mass Spectrum of SC2 (A) Mass Fragmentation patterns obtained for SC2 were suggestive of rosmarinic acid. (B) ^13^C NMR spectra of SC2. (C) Table summarising chemical shifts of both ^13^C NMR and ^1^H NMR spectra for SC2. ^13^C NMR and ^1^H NMR shifts and proposed structure of SC2 were highly suggestive of rosmarinic acid. (D) The proposed structure of SC2 (rosmarinic acid).

**Figure S3.** Mass Spectrum of SC1 (A) Mass Fragmentation patterns obtained for SC1 were suggestive of rosmarinic acid 4-O-β-d-glucoside (Rosmarinyl glucoside). (B) ^13^C NMR spectra of SC1. (C) Table summarising chemical shifts of both ^13^C NMR, and ^1^H NMR. ^13^C NMR, and ^1^H NMR shifts were highly suggestive of rosmarinic acid 4-O-β-d-glucoside. and the (D) proposed structure of SC1 (rosmarinic acid 4-O-β-d-glucoside).

**Table S3:** MIC and MBC (mg/ml) of Sephadex LH-20 fractions (SCMEXBu) compared to crude methanol extract (MEX).

|  | **MRSA ATCC43300** | **MRSA ATCC33591** | **SA ATCC29213** | **SE ATCC 35984** | **MRSE NCTC11964** | **EF DZMZ25390** |
| --- | --- | --- | --- | --- | --- | --- |
|  | ***Mean Inhibitory Concentration (MIC) (mg/ml)*** | | | | | |
| SCMEX | 1 | 0.5 | 1 | 2 | 4 | 2 |
| SCMEXBu1 | > 4 | > 4 | > 4 | > 4 | > 4 | > 4 |
| SCMEXBu2 | > 4 | > 4 | > 4 | > 4 | > 4 | > 4 |
| SCMEXBu3 | > 4 | >4 | >4 | > 4 | > 4 | > 4 |
| SCMEXBu4 | > 4 | > 4 | > 4 | > 4 | > 4 | > 4 |
| SCMEXBu5 | 0.125 | 0.125 | 0.25 | 0.25 | 0.5 | 0.125 |
| SCMEXBu6 | > 4 | > 4 | > 4 | > 4 | > 4 | > 4 |
| SCMEXBu7 | > 4 | > 4 | > 4 | > 4 | > 4 | > 4 |
|  | ***Mean Bactericidal Concentration (MBC) (mg/ml))*** | | | | | |
| SCMEX | 0.5 | 0.5 | 1 | 2 | 2 | > 4 |
| SCMEXBu1 | > 4 | > 4 | > 4 | > 4 | > 4 | > 4 |
| SCMEXBu2 | > 4 | > 4 | > 4 | > 4 | > 4 | > 4 |
| SCMEXBu3 | > 4 | >4 | >4 | > 4 | > 4 | > 4 |
| SCMEXBu4 | > 4 | > 4 | > 4 | > 4 | > 4 | > 4 |
| SCMEXBu5 | 0.25 | 0.25 | 0.5 | 1 | 1 | 0.25 |
| SCMEXBu6 | > 4 | > 4 | > 4 | > 4 | > 4 | > 4 |
| SCMEXBu7 | > 4 | > 4 | > 4 | > 4 | > 4 | > 4 |

**MRSA:** Methicillin-Resistant *Staphylococcus aureus*, **SA:** *Staphylococcus aureus*, **EF:** *Enterococcus faecalis* and **SE:** *Staphylococcus epidermidis*.
